# Hsa_circ_0051079 functions as an oncogene by regulating miR-26a-5p/TGF-β1 in osteosarcoma

**DOI:** 10.1186/s13578-019-0355-2

**Published:** 2019-11-29

**Authors:** Zuojun Zhang, Ming Zhao, Guojie Wang

**Affiliations:** 1 Upper Limb Injury Treatment Center, Luoyang Orthopedic Hospital of Henan Province, Luoyang, China; 2 Luoyang Orthopedic Hospital of Henan Province, Orthopedic Hospital of Henan Province, 82 Qiming South Road, Chanhe District, Luoyang, 122400 Henan China

**Keywords:** Hsa_circ_0051079, miR-26a-5p, TGF-β1, Osteosarcoma

## Abstract

**Background:**

Osteosarcoma is a most common bone malignant tumor which threatens children and adolescents. Circular RNAs (circRNAs) fundamentally play essential roles in the progress and development of human cancers by sponging with microRNAs (miRNAs). However, the role of circRNAs in osteosarcoma is not clear. The aim of the study was to investigate the roles and molecular mechanism of circRNAs in osteosarcoma.

**Results:**

The data from qRT-PCR showed that circ_0051079 expression was higher in osteosarcoma cells and tissues compared to their normal controls. Meanwhile, bioinformatic analysis indicated that circ_0051079 was a sponge of miR-26a-5p, which was verified by luciferase activity assay. Subsequently, TGF-β1 was verified as a putative target mRNA of miR-26a-5p by luciferase assay. Cellular function assays were conducted and the findings revealed that circ_0051079/miR-26a-5p/TGF-β1 regulated osteosarcoma proliferation and metastasis.

**Conclusion:**

The study demonstrated that circ_0051079 could act as an oncogene via regulating miR-26a-5p/TGF-β1 and a potential biomarker for osteosarcoma diagnose.

## Background

Osteosarcoma is regarded as the most common devastating malignant bone cancer in children and adolescents [1, 2] and results in high morbidity and mortality [3]. Presently, the main therapies for osteosarcoma are surgery, adjuvant chemotherapy and radiotherapy, which increase the survival rates, but those conventional therapies still limit its efficacy [4, 5]. The overall survival rate of osteosarcoma patients remains dismal due to the metastases and relapse [6]. So, it is urgent for scientists to discover in depth the molecular mechanisms underlying osteosarcoma progression and identify some relevant biomarkers for diagnosis and prognosis for patients.

Circular RNAs (circRNAs) are novel of endogenous non-coding RNAs which were firstly found in 1976 and considered as the byproducts of splicing errors with a low expression level [7, 8]. Recently, with the development of high-throughput sequencing and novel computational approaches, circRNAs are identified to play critical roles in regulating gene expression at transcriptional and post-transcriptional levels [8]. Many studies have reported the functions of circRNAs including mediating alternative splicing [9], interacting with proteins [10], and acting as microRNAs (miRNAs) sponges [11, 12]. Specially, circRNAs can serve as competing endogenous RNA (ceRNA) to inhibit miRNAs activity and further regulate the down-stream gene expression. So, many circRNAs were reported to take part in the cancer progression and function as biomarkers for the cancer diagnosis and prognosis [13]. Increasing evidence has demonstrated that circRNAs were revealed in many malignant tumors to regulate cell proliferation, migration, invasion and apoptosis. For instance, hsa_circ_0008039 exerted oncogenic roles in breast cancer [14]. Hsa_circ_0000673 exerted tumor-suppressing effects and served as a promising diagnosis biomarker and a therapeutic target in gastric cancer [15]. circ_0001649 could inhibit lung cancer cells growth and metastasis [16]. However, whether and how circRNAs control the development of osteosarcoma is unclear.

In the current study, it was found circ_0051079 was up-regulated in osteosarcoma by circRNA array and the data of cellular functions verified that circ_0051079 played an important role in osteosarcoma development and could function as an oncogene. The investigation in exploring the molecular mechanism identified that circ_0051079 sponged with miR-26a-5p to increase TGF-β1 expression. The study revealed that circ_0051079 could function as an oncogene in osteosarcoma.

## Results

### CircRNA expression in osteosarcoma tissues

**Fig. 1:**
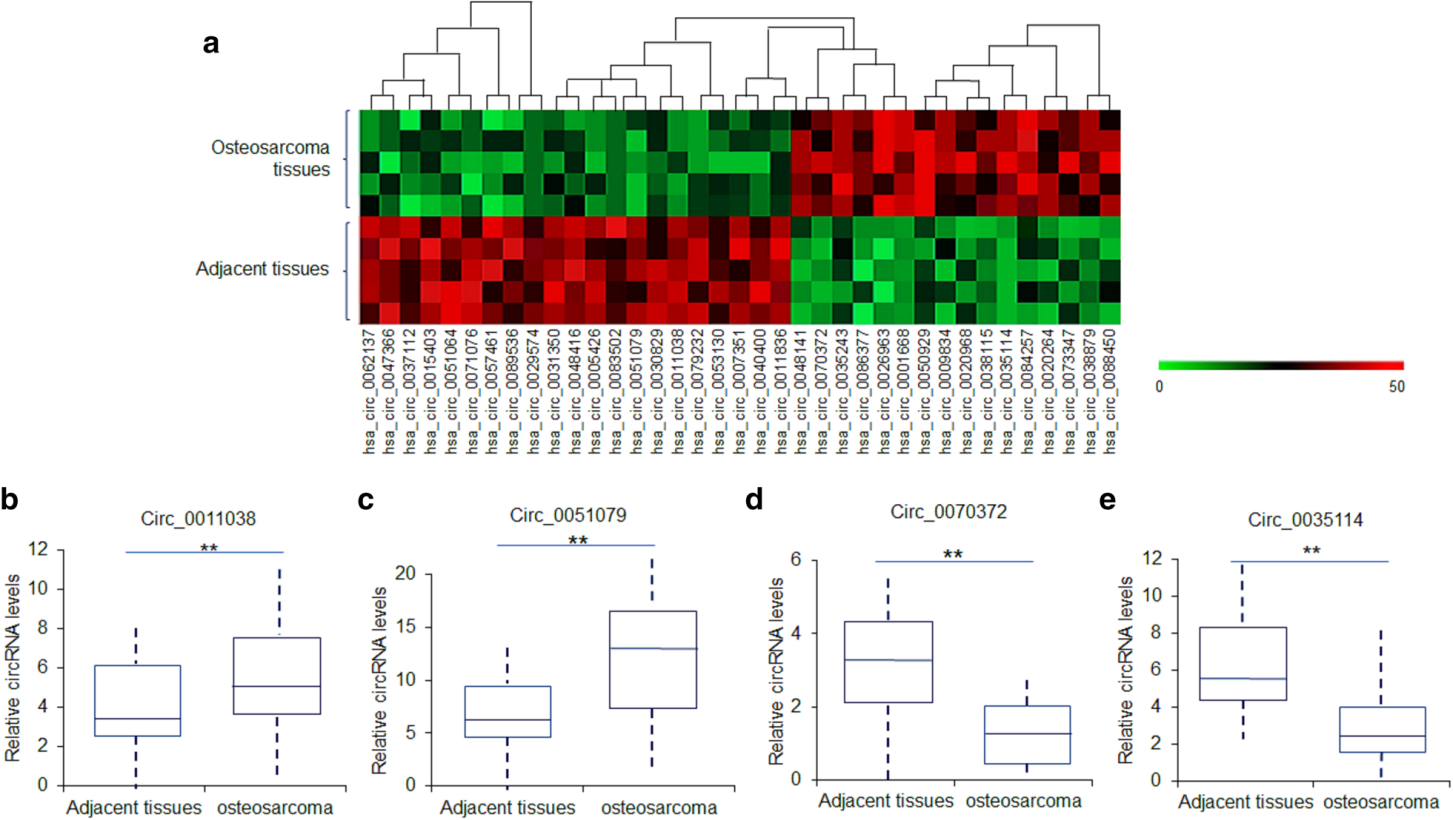
Deregulated circRNAs in osteosarcoma tumor tissues. **a** The heat map of significant circRNAs in osteosarcoma tissues and the matched non-tumor tissues. circRNAs were analyzed by circRNAs Arraystar Chip. The samples were from 5 osteosarcoma patients. **b**–**e** Relative expression of the four circRNAs from 45 osteosarcoma tumor tissues and adjacent non-tumor tissues listed in (**a**) measured by RT-qPCR. **p < 0.01

To explore the different circRNA profiles between osteosarcoma tissues and adjacent tissues, unsupervised hierarchical clustering was performed to visualize the differential circRNAs. The heatmap showed the top fifteen most increased and decreased circRNAs in osteosarcoma tissues and the matched non-tumor tissues by circRNAs Arraystar Chip (Fig. 1a). Two significant up-regulated and two down-regulated circRNAs were selected to validate in 45 osteosarcoma tumor tissues and adjacent non-tumor tissues by real-time quantitative RT-PCR. The levels of circ_0051079 and circ_0011038 expression were significantly higher in osteosarcoma tissues than their adjacent normal tissues (Fig. 1b, c). The two down-regulated circRNAs including circ_0070372 and circ_0035114 were lower in osteosarcoma tissues than in their adjacent normal tissues (Fig. 1d, e). The circ_0051079 levels were changed significantly and might be an oncogene, so it was chosen for the further investigation.

### Circ_0051079 was upregulated in osteosarcoma and correlated with poor survival ability

**Fig. 2:**
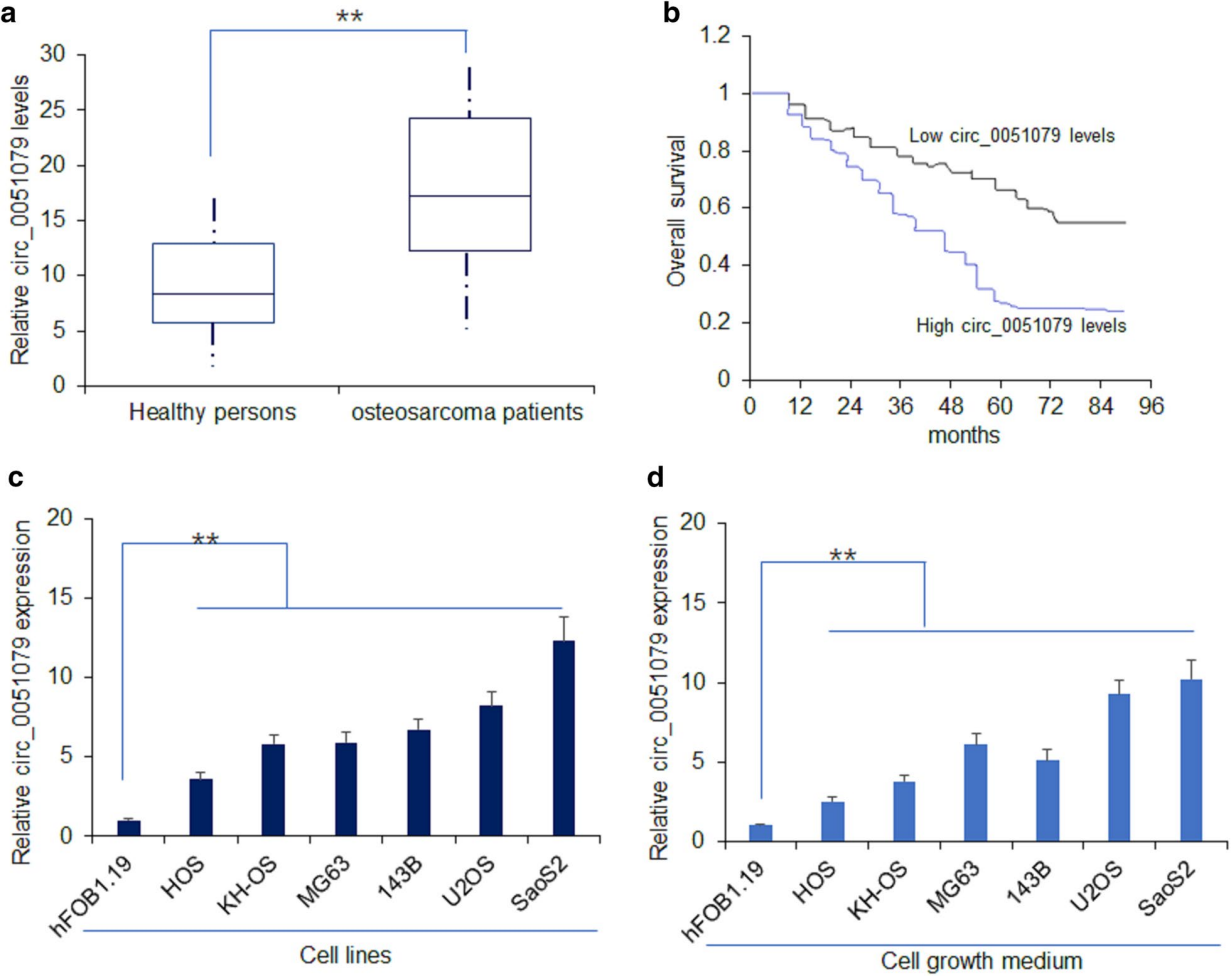
Circ_0051079 was upregulated in osteosarcoma patients and correlated with poor survival. **a** The expression level of circ_0051079 was detected in serum samples from osteosarcoma patients (n = 32) and healthy individuals (n = 32). **b** Kaplan–Meier curves for overall survival. There were 105 patients including 63 males and 42 females. The average age was 36.2 years old. **c** The expression level of circ_0051079 in six osteosarcoma cell lines and one normal osteoblastic cell line hFOB. **d** The expression level of circ_0051079 in the growth medium from six osteosarcoma cell lines and one normal osteoblastic cell line hFOB. **p < 0.01

Circ_0051079 was confirmed as a significant up-regulated circRNA and selected to explore its function in osteosarcoma. Firstly, we detected the circ_0051079 expression level in serum samples from osteosarcoma patients (n = 32) and healthy individuals (n = 32). The data showed that the expression of circ_0051079 up-regulated greatly in the blood samples from osteosarcoma patients compared to the healthy individuals (Fig. 2a). At the same time, we found that there was a negative relationship between the overall survival and circ_0051079 expression levels. Patients with high circ_0051079 levels have a shorter survival time than the patients with low circ_0051079 levels (Fig. 2b). Subsequently, the circ_0051079 expression was performed in six osteosarcoma cell lines (HOS, KH-OS, MG63, 143B, U2OS, SaoS2) and one normal osteoblastic cell line (hFOB). Figure 2c showed that the circ_0051079 expression was significantly up-regulated in HOS, KH-OS, MG63, 143B, U2OS, SaoS2 cells compared to hFOB cells. Remarkably, the expression levels of circ_0051079 in the growth medium were also higher in HOS, KH-OS, MG63,143B, U2OS, SaoS2 cells compared to hFOB cells (Fig. 2d). Therefore, the results demonstrated that circ_0051079 was up-regulated in osteosarcoma and had a poor correlation with the overall survival.

### Circ_0051079 negatively regulated miR-26a-5p in osteosarcoma cells

**Fig. 3:**
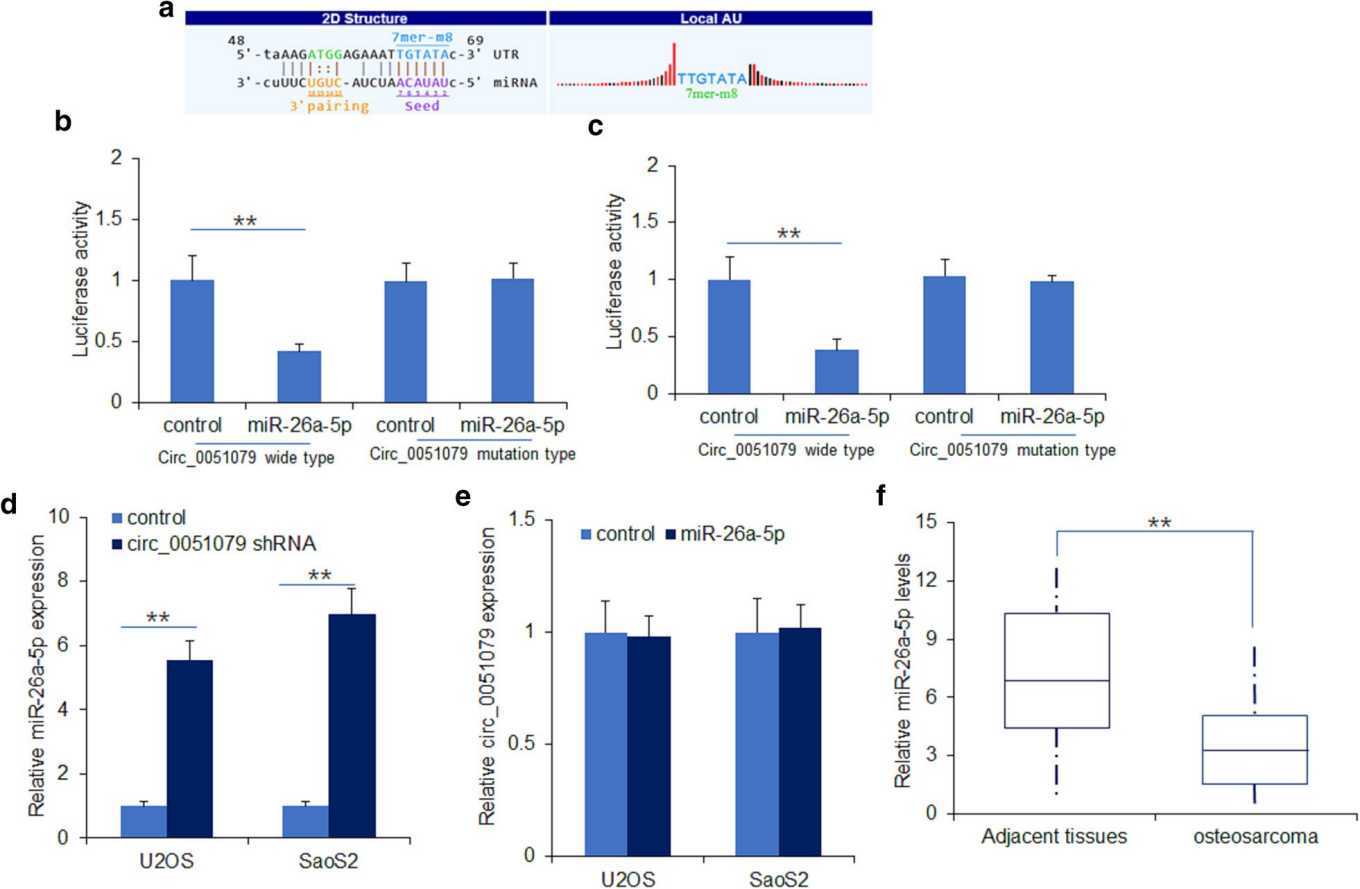
Circ_0051079 negatively regulated miR-26a-5p in osteosarcoma cells. **a** The putative sequences of miR-26-5p and circ_0051079 binding site. **b**, **c** Luciferase activity of circ_0051079 in osteosarcoma cells. Cells were transfected with miR-26a-5p and the plasmids with the circ_0051079 reporter and then luciferase activity was analyzed by dual-luciferase system. **d** MiR-26a-5p expression in osteosarcoma cells. The cells were transfected with circ_0051079 shRNA for 48 h and the levels of it was detected by real time RT-PCR. **e** Circ_0051079 expression in osteosarcoma cells. The cells were transfected with miR-26a-5p mimics for 48 h and the levels of it was detected by real time RT-PCR. **f** MiR-26a-5p expression in osteosarcoma tissues. Total RNA from 32 primary osteosarcoma tissues was extracted for miR-26a-5p examination by real time RT-PCR. **p < 0.01

CircRNAs regulate the target gene expression by sponging with miRNAs. To further explore the underlying mechanism of circ_0051079 in osteosarcoma, targetscan and miRanda were used to search the potential targets of circ_0051079. Bioinformatics analysis predicted that circ_0051079 was the potential sponge of miR-26a-5p (Fig. 3a). Luciferase reporter assay was conducted to determine the reliability. The result illustrated that upregulation of miR-26a-5p notably decreased the luciferase activity of circ_0051079 wild-type, but had no effect on circ_0051079 mutant-type (Fig. 3b, c). Moreover, circ_0051079 shRNAs were transfected to osteosarcoma cells (U2OS and SaoS2) and miR-26a-5p expression was detected by qRT-PCR. We found that down-regulation of circ_0051079 decreased the expression of miR-26a-5p in the osteosarcoma cells (Fig. 3d). However, overexpression of miR-26a-5p does not alter the expression level of circ_0051079 (Fig. 3e). Furthermore, the miR-26a-5p expression was detected in osteosarcoma tissues. The result revealed that miR-26a-5p was down-regulated in 45 primary osteosarcoma tissues in contrast to paired adjacent normal tissues (Fig. 3f). These results proved that circ_0051079 might bind to miR-26a-5p in osteosarcoma cells.

### Circ_0051079 played an oncogenic role in osteosarcoma cells via miR-26a-5p

**Fig. 4:**
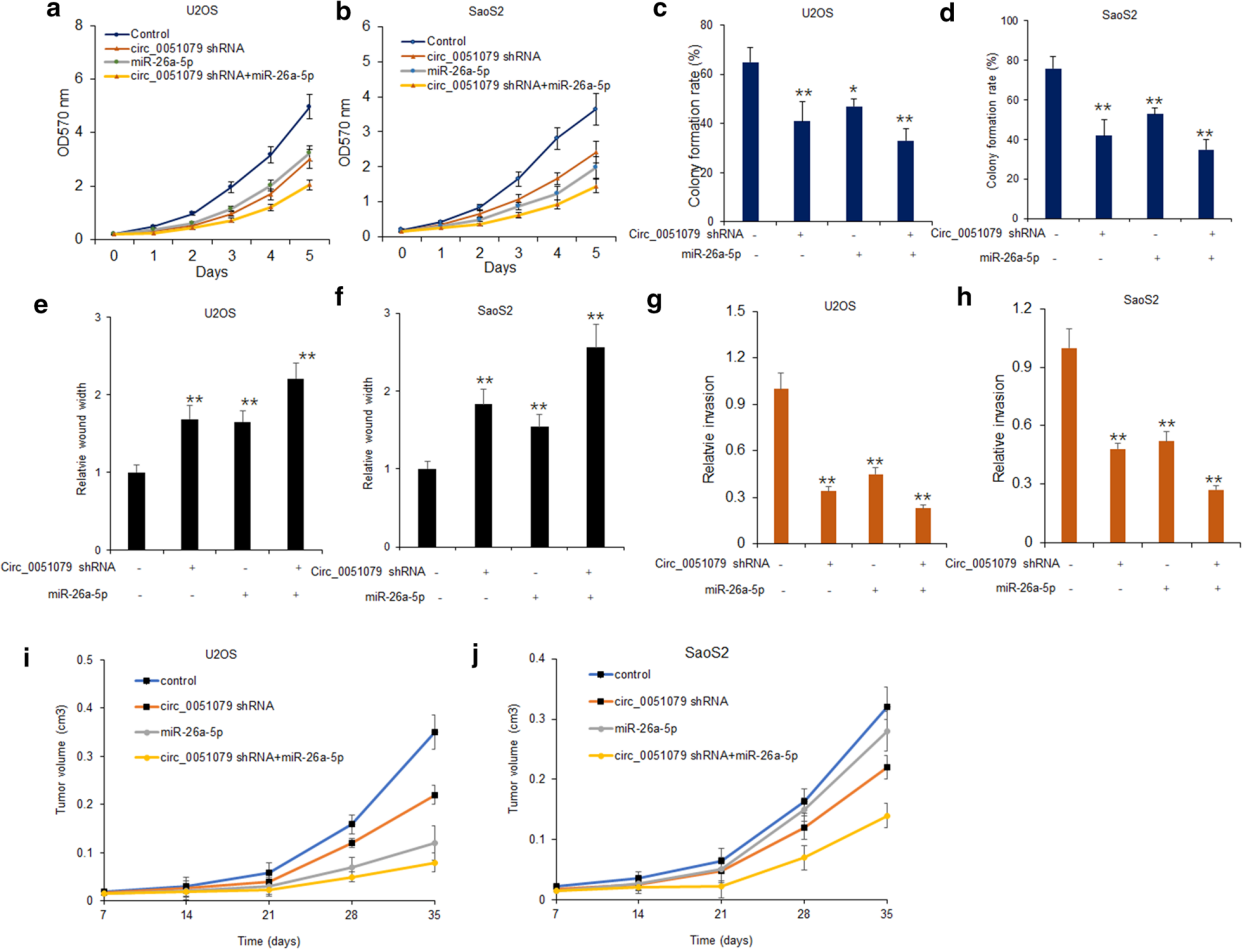
Circ_0051079 played an oncogenic role in osteosarcoma cells via miR-26a-5p. **a**, **b** Cell proliferation ofU2OS and SaoS2 cells. U2OS and SaoS2 cells were transfected with circ_0051079 shRNA or miR-26-5p mimics for 24 h and then MTT assay was used to evaluate the cell proliferation of U2OS and SaoS2 cells. **c**, **d** Cell proliferation of U2OS and SaoS2 cells. U2OS and SaoS2 cells were transfected with circ_0051079 shRNA or miR-26-5p mimics for 24 h and then cell colony formation assay was used to evaluate the cell proliferation of U2OS and SaoS2 cells. **e**, **f** The migratory ability of U2OS and SaoS2 cells. U2OS and SaoS2 cells were transfected with circ_0051079 shRNA or miR-26a-5p mimics for 24 h and cell migration was analyzed. **g**, **h** The invasion ability of U2OS and SaoS2 cells. U2OS and SaoS2 cells were transfected with circ_0051079 shRNA or miR-26a-5p mimics for 24 h and cell migration was analyzed. **i**, **j** The growth curve of U2OS and SaoS2 cells in vivo. U2OS and SaoS2 cells with circ_0051079 shRNA or miR-26a-5p mimics were injected to the nude mice and the tumor size was recorded and analyzed every 7 days. **p < 0.01

To deeply study the function of circ_0051079 in osteosarcoma cells, circ_0051079 shRNA and/or miR-26a-5p was introduced into osteosarcoma cells (U2OS and SaoS2). MTT assay was used to observe the proliferation of U2OS and SaoS2. The data showed that cell proliferation was dramatically inhibited by knocking-down of circ_0051079 or up-regulation of miR-26a-5p in U2OS and SaoS2 cells respectively (Fig. 4a, b). The result of colony formation assay indicated that the colonies remarkably decreased in U2OS and SaoS2 cells with suppressing circ_0051079 expression and/or over-expressing miR-26a-5p expression (Fig. 4c, d). Similarly, we detected the migration and invasion ability in U2OS and SaoS2 cells transfected with circ_0051079 shRNA or miR-26a-5p mimics. Wound-healing assay indicated that circ_0051079 inhibition and/or miR-26a-5p over-expression could effectively decrease the migration ability in U2OS and SaoS2 cells (Fig. 4e, f and Additional file 1: Figure S2). At the same time, the invasion assay also proved that the silence of circ_0051079 and/or up-regulation of miR-26a-5p could inhibit the invasion ability of U2OS and SaoS2 cells (Fig. 4g, h and Additional file 1: Figure S2). Furthermore, the in vivo data indicated that inhibiting circ_0051079 expression led to tumor growth suppression (Fig. 4i, j). Thus, these experiments in vitro and in vivo demonstrated that circ_0051079 silencing inhibited the proliferation, migration and invasion by regulating miR-26a-5p, suggesting the oncogenic role of circ_0051079 in osteosarcoma cells via miR-26a-5p.

### MiR-26a-5p targeted TGFB1 in osteosarcoma cells

**Fig. 5:**
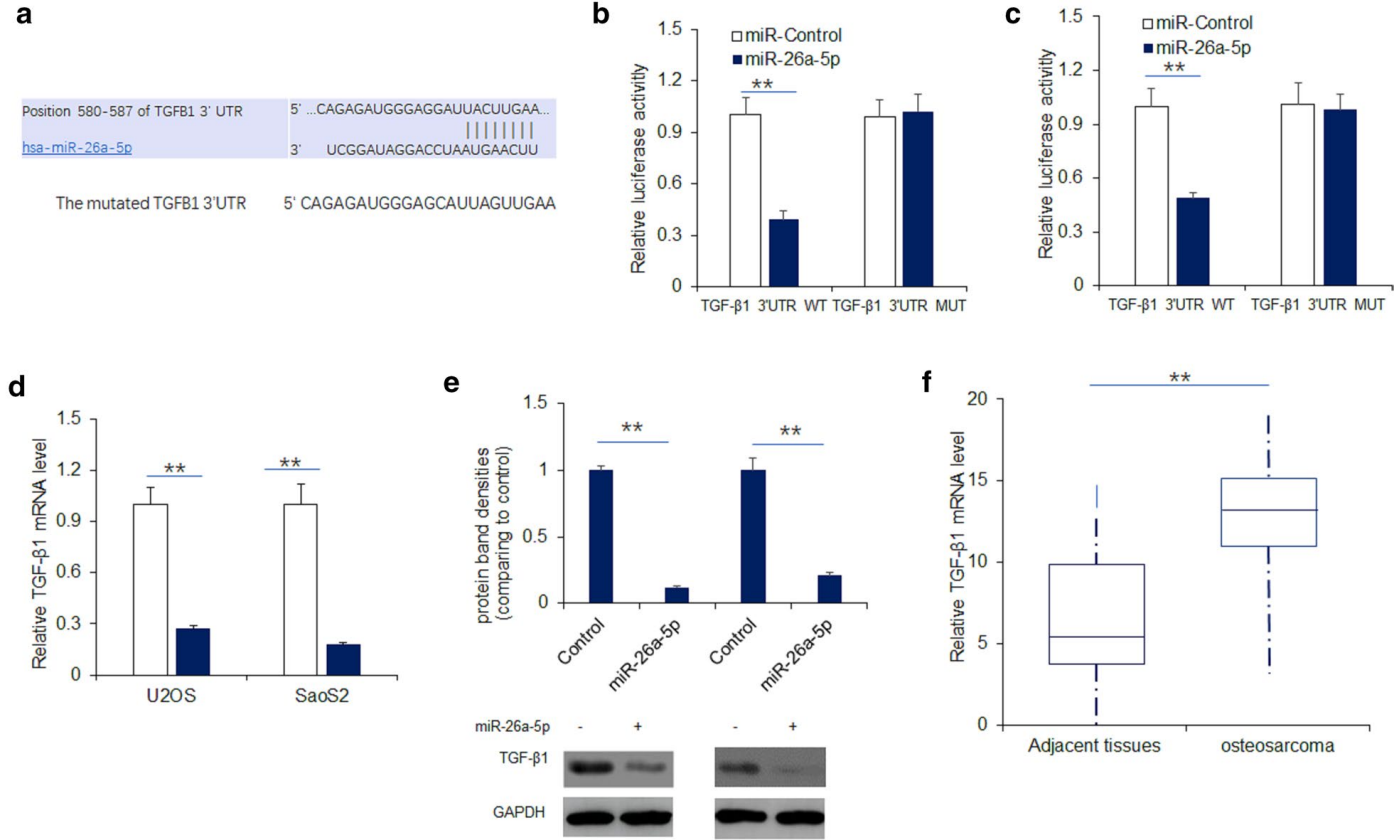
MiR-26a-5p suppressed osteosarcoma cell proliferation and invasion by targeting TGFB1 expression. **a** The putative sequences of miR-26a-5p and TGF-β1 with a binding site. **b**, **c** The luciferase activity of TGF-β1 3′UTR. U2OS and SaoS2 cells were transfected with plasmids containing wild type reporter for TGF-β1, or mutated reporter for TGF-β1 or miR-26a-5p and then luciferase activity was analyzed by dual luciferase system. **d**, **e** TGF-β1 mRNA and protein levels in U2OS and SaoS2 cells with miR-26a-5p up-regulation. U2OS and SaoS2 cells were transfected with miR-26a-5p and total RNA or protein was extracted for real time RT-PCR or Western blotting respectively. **f** Total RNA was extracted from osteosarcoma tissues for TGF-β1 mRNA detection by real time RT-PCR. The adjacent tissues were the controls. **p < 0.01

Bioinformatics analysis was applied to predict the binding site between miR-26a-5p and TGF-β1 (Fig. 5a). Subsequently, luciferase reporter assay confirmed that TGF-β1 was targeted by miR-26a-5p. Over-expression of miR-26a-5p effectively inhibited the luciferase activity of TGF-β1-WT (wild-type), but made no difference to TGF-β1-MUT (mutant-type) in both U2OS and SaoS2 cells (Fig. 5b, c). Then, we built a miR-26a-5p over-expression cell models by transfecting miR-26a-5p overexpression vector. The qRT-PCR and western blotting result found that miR-26a-5p overexpression significantly suppressed TGF-β1 mRNA and protein levels (Fig. 5d, e). In osteosarcoma tissues, TGF-β1 mRNA level was higher than adjacent tissues (Fig. 5f). In summary, we confirmed miR-26a-5p targeted TGF-β1 in osteosarcoma cells.

### Circ_0051079 promoted osteosarcoma progression by miR-26a-5p/TGF-β1

**Fig. 6:**
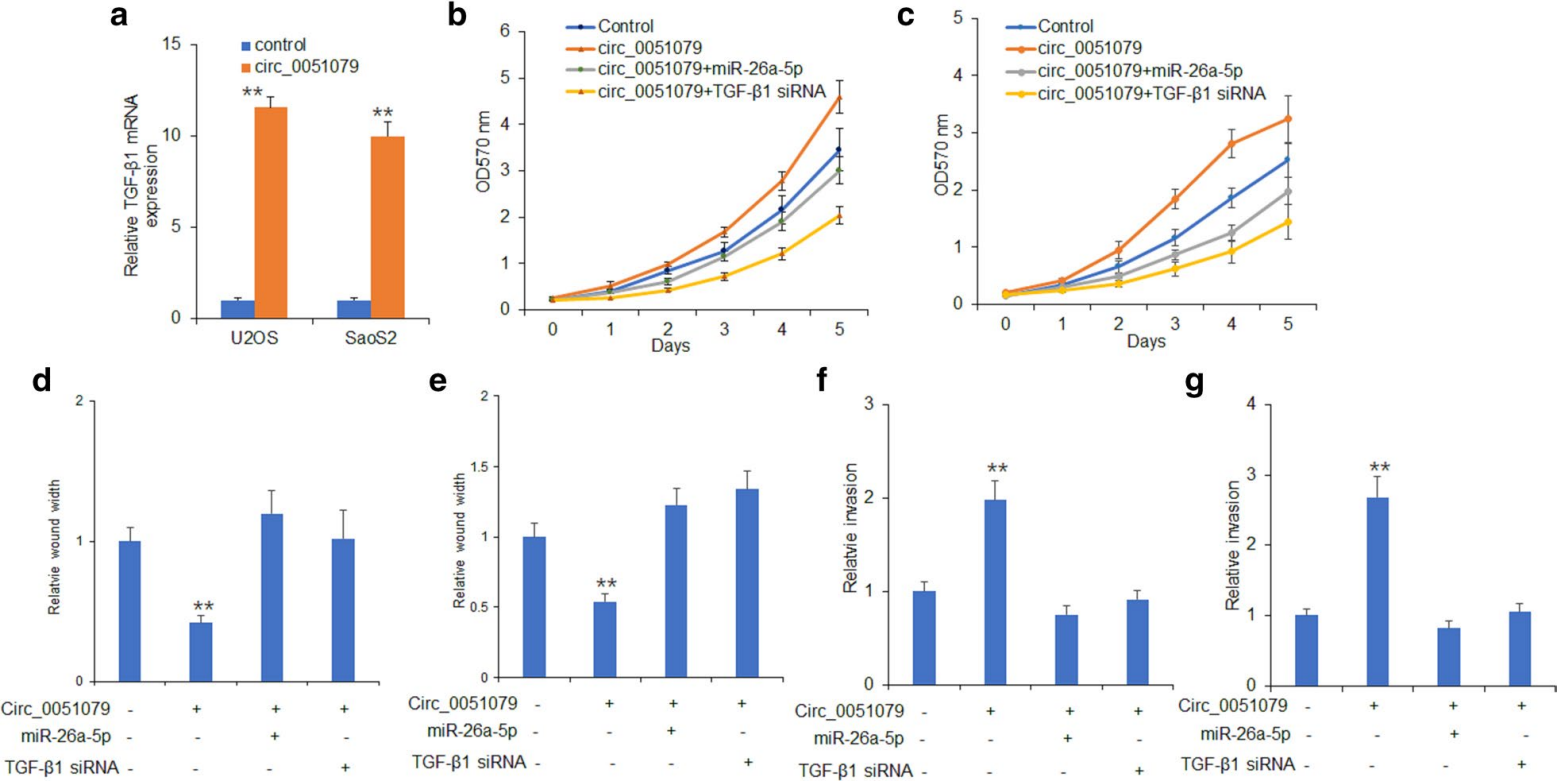
Circ_0051079 promoted osteosarcoma cell proliferation and metastasis through miR-26a-5p/TGF-β1. **a** TGFB1 mRNA and protein levels in U2OS and SaoS2 cells. U2OS and SaoS2 cells were transfected with circ_0051079 plasmids and circ_0051079 levels were assayed by real time RT-PCR. **b**, **c** Cell proliferation in U2OS and SaoS2 cells. U2OS and SaoS2 cells were transfected with circ_0051079, miR-26a-5p mimics or TGF-β1 shRNAs for 24 h and then cell proliferation was assayed by MTT. **d**, **e** Cell migration in U2OS and SaoS2 cells. U2OS and SaoS2 cells were transfected with circ_0051079, miR-26a-5p mimics or TGF-β1 shRNAs for 24 h and then cell migration was assayed by transwell chamber. **f**, **g** Cell invasion in U2OS and SaoS2 cells. U2OS and SaoS2 cells were transfected with circ_0051079, miR-26a-5p mimics or TGF-1 shRNAs for 24 h and then cell invasion was assayed by transwell system. **p < 0.01

To clarify the underlying mechanism of circ_0051079 in osteosarcoma, the overexpressed circ_0051079 vector was transfected to U2OS and SaoS2 cells. TGF-β1 mRNA level was up-regulated along with circ_0051079 overexpression (Fig. 6a). MTT assay showed that circ_0051079 remarkably promoted osteosarcoma cells proliferation, however, this effect was significantly abrogated by co-transfection with miR-26a-5p mimics or TGF-β1 siRNAs (Fig. 6b, c). Consistently, the enhanced cell invasive and migrant capacity of both cell lines induced by circ_0051079 was abolished by co-transfection with miR-26a-5p mimics or TGF-β1 siRNA (Fig. 6d–g). In addition, when the cells were transfected with circ_0051079 shRNA, the TGF-β1 signaling pathway was inhibited (Additional file 1: Figure S2). The relationship from the clinic data analysis showed that there was a positive relationship between circ_0051079 and TGF-β1 expression in osteosarcoma (Additional file 1: Figure S3). Overall, these results showed that circ_0051079 promoted osteosarcoma progression by sponging miR-26a-5p which suppressed TGF-β1 expression.

## Discussion

In clinic, there are great obstacles to treat osteosarcoma. It is necessary to find new target molecules for the early diagnosis and therapy of osteosarcoma. To better learn the roles of circRNAs in osteosarcoma, circRNA array was performed to find the different circRNAs in osteosarcoma. Circ_0051079 was verified as a significant up-regulated circRNA in osteosarcoma patients and associated with a short survival time. Circ_0001564 was found to regulate osteosarcoma proliferation and apoptosis [17]. The investigation of the underlying mechanism of circ_0051079 in osteosarcoma showed that it was sponged with miR-26a-5p and regulated osteosarcoma cell growth and metastasis by targeting TGF-β expression.

MiRNAs are attached to non-coding RNA and responsible to cellular and physiological processes by targeting protein-coding genes at post-transcriptional levels [18, 19]. MiR-26a-5p, locating on the human chromosome 3p22 [20], was the targeted downstream gene which predicted by target scan and functions in the development, differentiation, and cycle regulation for many cancers. For example, Xu et al. demonstrated that miR-26a-5p potentiated lung cancer cell metastasis via JAK2/STAT3 pathway by targeting ITGb8 [19]. Wang et al. found that miR-26a-5p regulated hepatoblastoma development by restraining the LIN28B-RAN-AURKA pathway [21]. Milena Rizzo et al. demonstrated that the re-expression of miR-26a-5p in DU-145 prostate tumor cells reduced the proliferation [22]. In the present study, miR-26a-5p was proved to the target bindings of circ_0051079 by dual-luciferase activity assay. Then, a series of experiments in vitro indicated that miR-26a-5p over-expression could inhibit osteosarcoma cells proliferation, migration and invasion. So, it was suggested that miR-26a-5p could function as anosteosarcoma suppressor. Furthermore, we found that TGF-β1 was a direct target gene of miR-26a-5p and involved in osteosarcoma progression and development.

TGF-β is a multifunctional growth factor involved in cell proliferation, differentiation, and extracellular matrix protein synthesis [23]. TGF-β1 encodes a secreted ligand of TGF-β that could bind various TGF-β receptors leading to recruitment and activation of SMAD family transcription factors that regulate gene expression. According to many reports, TGF-β1 plays a critical role in tumorigenesis as well as metastasis within tumor progression and development [24–29]. TGF-β1 extremely exerted essential role in many aspects of cancers [24–29], but the specific function and mechanism of TGF-β1 was not elucidated in osteosarcoma. In current study, we confirmed that miR-26a-5p could interact with 3′-untranslated regions (3′-UTR) of TGF-β1 mRNAs through luciferase activity assay. Meanwhile, the mRNA level and protein level of TGF-β1 were found to be higher in osteosarcoma cell lines and tissues compared with normal one. Thus, we confirmed that circ_0051079 promoted osteosarcoma progression by sponging miR-26a-5p and upregulation of TGF-β1.

## Conclusions

Circ_0051079 was up-regulated in osteosarcoma and correlated with poor survival time. Circ_0051079 was verified as a sponge of miR-26a-5p. Furthermore, miR-26a-5p targeted TGF-β1 to involve in the regulation of osteosarcoma. In brief, circ_0051079/miR-26a-5p/TGF-β1 regulated osteosarcoma progression and development. Our findings revealed that circ_0051079 could act as an oncogene and a potential biomarker for osteosarcoma.

## Methods

### Osteosarcoma samples

Total 45 osteosarcoma tissues and adjacent normal tissues were collected from Luoyang Orthopedic Hospital of Henan Province (Zhengzhou, China) between 2013 and 2016. The samples from the healthy individuals were from the healthy peoples without any diseases. The blood samples from osteosarcoma patients and healthy individuals were collected for use. All samples were instantly snap-frozen and stored at − 80 °C until use. Informed contents were written and obtained from all patients (Table 1).

**Table 1:** The information of patients with osteosarcoma

Characteristics	Numbers
Gender	
Male	27
Female	18
Age (years)	
< 40	32
≥ 40	13
Tumor size (cm)	
< 6	29
≥ 6	16
WHO grade	
I–II	31
III	14

### Cell culture

Six human osteosarcoma cell lines (HOS, KH-OS, MG63, 143B, U2OS and SaoS2) and one normal osteoblast cell line (hFOB1.19) were all purchased from American Type Culture Collection (ATCC). They were cultured in Dulbecco’s Modified Eagle’s medium (DMEM) (Gibco, Gaithers-burg, MD, USA) growth medium with 10% fetal bovine serum (Gibco), 50 μg/ml of penicillin and 50 μg/ml of streptomycin at 37 ℃ containing a humidified atmosphere of 5% CO_2_.

### Cell transfection

Lipofectamine 2000 (Invitrogen) was used to transfect recombinant vectors or control vectors into U2OS and SaoS2 cells according to the manufacturer’s instruction. The recombinant vectors included circ_0051079, circ_0051079 shRNA, miR-26a-5p, miR-26a-5p mimics or TGF-β1 siRNA (Santa Cruz). All the vectors were constructed by Hanheng Biotech (Shanghai, China). The sequences were covered by a patent. About 2 × 10^5^ cells were planted in six well plates. The recombinant vector was transfected after 24 h. The efficiency of knockdown and up-regulation was analyzed using qRT-PCR.

### RNA extraction and real-time PCR analysis

Total RNA was extracted from sample tissues and cells using TRIzol regent (Invitrogen, Carlsbad, CA, USA) according to the manufacturer’s specification. Total RNA was reverse transcribed with Prime Script TM RT reagent Kit with gDNA Eraser (TaKaRa, Dalian, Liaoning, China). The quantitative RT-PCR was performed with SYBR- Green PCR Master Mix kit (Takara, Japan) on a StepOne Plus real time PCR system (Applied Biosystems, Foster City, CA, USA). Glyceraldehyde 3-phosphate dehydrogenase (GAPDH) was an internal control and U6 was used as the control of miR-26a-5p based on the comparative Ct method (2^−ΔΔCt^). The primer sequences used for qRT-PCR and the sequence of miR-26a-5p were as the following:

TGF-β1

Forward Primer: 5′GGCCAGATCCTGTCCAAGC3′

Reverse Primer: 5′GTGGGTTTCCACCATTAGCAC3′

Circ_0051079

Forward Primer: 5′TTTGGCAAAGTCATCCTGGT3′

Reverse Primer: 5′TGGTACGCTGTCACCTAGCTC3′

Circ_0011038

Forward Primer: 5′GAGAAAGGCTGGGTCTTGG3′

Reverse Primer: 5′AGTCAAGGCTGCCGTTCTT3′

Circ_0070372

Forward Primer: 5′CAGCAGCAGAAAGTGGAAAA3′

Reverse Primer: 5′ACTGCCAGTGCTGATTGCT3′

Circ_0035114

Forward Primer: 5′TGCGAGAAACCTTCCTCAAC3′

Reverse Primer: 5′GGAGCAGCTCTAGCCAGGAT3′

miR-26a-5p.1

Forward Primer: 5′GCAGTTCAAGTAATCCAGGATAG3′

Reverse Primer: 5′GTCGTATCCAGTGCAGGGTCCGAGGTATTCGCACTGGATACGAC3′.

### Microarray analysis

After being obtained from surgical specimens, samples were immediately frozen using liquid nitrogen. Sample preparation and microarray hybridization were performed according to the protocols of Arraystar (Rockville, MD, USA). CircRNAs were enriched through removing linear RNAs with Rnase R (Epicentre, Madison, WI, USA), and then amplified and labelled using Arraystar Super RNA Labeling Kit (Arraystar). mRNAs were purified using rRNA removal kit (Arraystar). Subsequently, Arraystar Human circRNA Array (8 × 15K) and LncPath Human Cancer Array were used for hybridization, and then scanned by the Agilent Scanner G2505C (Jamul, CA, USA). circRNAs and mRNAs demonstrating fold-changes of ≥ 2 and p-values of less than 0.05 were regarded as significantly differentially expressed.

### ceRNA analysis and target prediction

We constructed a hsa_circRNA_100290-miRNA-target gene network using Cytoscape to visualize their interactions based on our circRNA microarray data and mRNA microarray data. In the network, we predicted the circRNA/miRNA interaction with miRNA target prediction software (Arraystar’s home-made) established from TargetScan and miRanda. The triple network was based on the theory of ceRNA that the circRNA shared the same miRNA with mRNA or other non-coding RNAs in one triplet.

### Cell proliferation

MTT (Sigma, USA) was performed for the cell proliferation analysis based on the manufacturer’s protocol. The transfected osteosarcoma cells were seeded in 96-well plates with 5 × 10^3^ cells in every well. The second day, 20 μl MTT solution (5 mg/ml) was added to cells per well incubated at 37 °C for 4 h. Then, abandon supernatant and add 150 μl DMSO (dimethylsulfoxide) to dissolve sediment. Lastly, the absorbance was measured at 570 nm through microplate reader (BioTek). All experiments were repeated at least three times independently.

### Nude mouse experiments

To study tumor growth, 5- to 6-week-old female, athymic nude BALB/c mice (Vital River Laboratory Animal Technology Co. Ltd., China) were injected with 2 × 106 U2OS and SaoS2 cells in 1 ml PBS. The mice were then divided into a circ_0051079 shRNA group and a control group with six mice in each group. At the day 7, 14, 21, 28, 35 following tumor cell injection, tumor size was measured and tumor growth was analyzed.

### Colony formation assay

The transfected osteosarcoma cells, about 1 × 10^3^, were planted in 6-well plate and cultured with DMEM completed growth medium at 37 °C for 14 days. Colonies were fixed with 4% paraformaldehyde and stained with 0.1% crystal violet (Beyotime, Shanghai, China) for 30 min. Then, wash cells and take photos. Colonies containing > 50 cells were manually counted. Each experiment was conducted in triplicate for more than three independent experiments.

### Wound healing assays

Osteosarcoma cells were seeded in six-well plates and cultured to 90% confluence under same culture condition. A plastic pipette tip was used to scratch the cell monolayer and generate wounds. Phosphate buffered saline (PBS) was used to rinse and remove the floating cells. The wounds distance was measured with Image pro plus 6.0 software (Media Cybernetics Inc., MD, USA) at 48 h. The data were representative of three individual experiments carried out in triplicate.

### Transwell assays

About 1 × 10^4^ transfected cells were added into the transwell chambers (Corning, USA) which coated with Matrigel (BD Bioscience, MA, USA) in upper surface. Growth medium without fetal bovine serum was added in the upper chamber and 600 μl medium containing 10% fetal bovine serum was added into the low chamber. After 24 h, the upper cells invade to bottom surface. The cells under the membrane were fixed and strained using 0.1% crystal violet. Three randomly selected fields were captured and calculate the number of cells that had success fully invaded and transmigrated the Matrigel with Image J software.

### Dual luciferase reporter assay

The binding sites were identified by JASPAR (http://jaspar.genereg.net/) within circ_0051079, miR-26a-5p and TGF-β1. The constructed luciferase reporter plasmid pLG4.0 (Promega, Madison, WI, USA) or empty vector was transfected into cells. The luciferase activity was measured using the dual-luciferase reporter kit (Promega, Madison, WI, USA) after transduction 48 h. The relative firefly luciferase activity was normalized to Renilla luciferase activity.

### Western blotting

The total protein of the osteosarcoma tissues and cells was harvested with cold RIPA (radio immunoprecipitation assay buffer). Bicinchoninic acid (BCA) protein assay kit (Pierce Biotechnology, Rockford, IL, USA) was performed to qualify the protein concentration. Then, equal protein samples were loaded into 10% SDS-PAGEand transferred onto a PVDF membrane, which was incubated in the primary antibodies and then the secondary antibodies. Antibodies to TGF-β1 and GAPDH used in this study were purchased from ABCAM.

### Statistical analysis

Data from three independent experiments were performed using the SPSS 15.0 statistical software (SPSS Incorporation, Chicago, IL) and emerged as mean ± standard deviation (SD). Difference between two groups was assessed using Student’s t test or one-way ANOVA. Kaplan–Meier method was used to analyze the survival curves for patients with high or low expression of circ_0051079. p < 0.05 was considered to be statistically significant.

## Supplementary information

**Additional file 1: Figure S1.** **a** The microscopic images of migration assays. The migratory ability of U2OS and SaoS2 cells. U2OS and SaoS2 cells were transfected with circ_0051079 shRNA or miR-26a-5p mimics for 24 h and cell wound healing assay was performed and the photos were taken under a microscope. **b** The invasion ability of U2OS and SaoS2 cells. U2OS and SaoS2 cells were transfected with circ_0051079 shRNA or miR-26a-5p mimics for 24 h and the photos of invaded cells were taken under a microscope. **Figure S2.** The effects of Hsa_circ_0051079 knockdown or overexpression on the protein expression of TGFB1 and the activation of TGFB signaling. The total protein from U2OS and SaoS2 cells were extracted for western blotting. **Figure S3.** The correlation between Hsa_circ_0051079 and TGF-β1 in patients.

## Data Availability

The datasets used and/or analyzed during the current study are available from the corresponding author on reasonable request.
