# Supplementary material for: Hsa_circ_0051079 functions as an oncogene by regulating miR-26a-5p/TGF-β1 in osteosarcoma
Source: Cell Biosci. 2019 Nov 29;9:94. doi: 10.1186/s13578-019-0355-2 (PMC6883546; doi:10.1186/s13578-019-0355-2)
Supplement: Supplementary file 1 — Additional file 1: Figure S1. a The microscopic images of migration assays. The migratory ability of U2OS and SaoS2 cells. U2OS and SaoS2 cells were transfected with circ_0051079 shRNA or miR-26a-5p mimics for 24 h and cell wound healing assay was performed and the photos were taken under a microscope. b The invasion ability of U2OS and SaoS2 cells. U2OS and SaoS2 cells were transfected with circ_0051079 shRNA or miR-26a-5p mimics for 24 h and the photos of invaded cells were taken under a microscope. Figure S2. The effects of Hsa_circ_0051079 knockdown or overexpression on the protein expression of TGFB1 and the activation of TGFB signaling. The total protein from U2OS and SaoS2 cells were extracted for western blotting. Figure S3. The correlation between Hsa_circ_0051079 and TGF-β1 in patients. [file 13578_2019_355_MOESM1_ESM.docx]

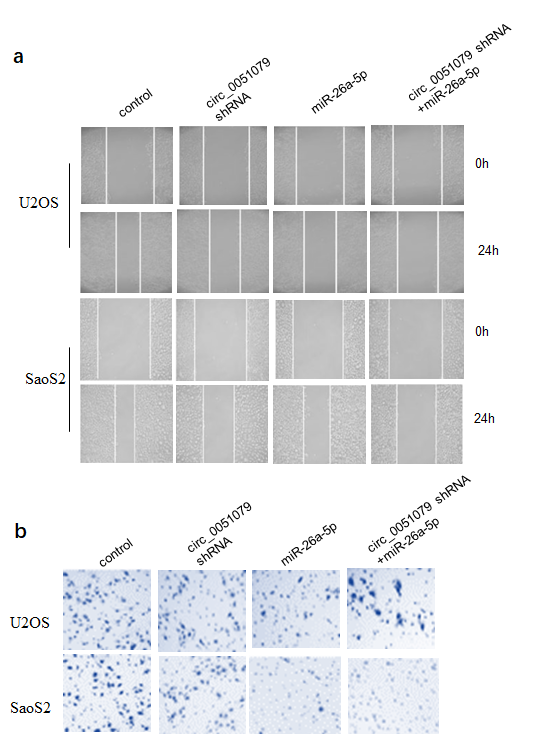


Additional file 1: Figure S1. **a** The microscopic images of migration assays. The migratory ability of U2OS and SaoS2 cells. U2OS and SaoS2 cells were transfected with circ_0051079 shRNA or miR-26a-5p mimics for 24h and cell wound healing assay was performed and the photos were taken under a microscope. **b** The invasion ability of U2OS and SaoS2 cells. U2OS and SaoS2 cells were transfected with circ_0051079 shRNA or miR-26a-5p mimics for 24h and the photos of invaded cells were taken under a microscope.


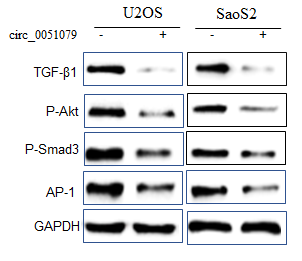


Additional file 1: Figure S2. The effects of Hsa_circ_0051079 knockdown or overexpression on the protein expression of TGFB1 and the activation of TGFB signaling. The total protein from U2OS and SaoS2 cells were extracted for western blotting.


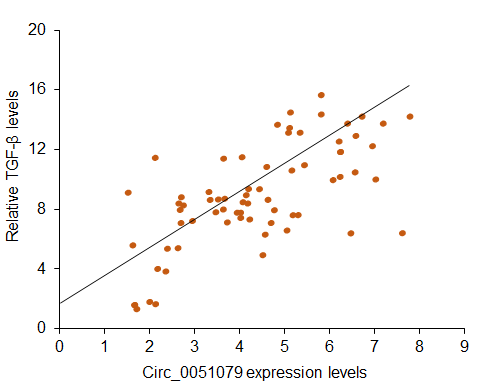


Additional file 1: Figure S3. The correlation between Hsa_circ_0051079 and TGF-β1 in patients.
